# A Checklist for Successful Quantitative Live Cell Imaging in Systems Biology

**DOI:** 10.3390/cells2020284

**Published:** 2013-04-29

**Authors:** Myong-Hee Sung

**Keywords:** quantitative microscopy, live cell imaging, fluorescent proteins, mathematical modeling, network models

## Abstract

Mathematical modeling of signaling and gene regulatory networks has provided unique insights about systems behaviors for many cell biological problems of medical importance. Quantitative single cell monitoring has a crucial role in advancing systems modeling of molecular networks. However, due to the multidisciplinary techniques that are necessary for adaptation of such systems biology approaches, dissemination to a wide research community has been relatively slow. In this essay, I focus on some technical aspects that are often under-appreciated, yet critical in harnessing live cell imaging methods to achieve single-cell-level understanding and quantitative modeling of molecular networks. The importance of these technical considerations will be elaborated with examples of successes and shortcomings. Future efforts will benefit by avoiding some pitfalls and by utilizing the lessons collectively learned from recent applications of imaging in systems biology.

## 1. Introduction

Here I will primarily focus on practical aspects of conducting live cell microscopy studies. For conceptual advantages of real time live cell monitoring over snapshot imaging of single cells or cell population studies, readers are referred to [1,2]. This essay was motivated by an increasing number of investigators who wish to benefit from live cell imaging but need guides to establish a successful system that meets the necessary requirements.

## 2. Considerations for Artifact-Free Monitoring in Live Cell Imaging

Although many tools are borrowed from techniques commonly used in molecular cell biology, special needs arise due to specific requirements of systems biology. Moreover, routinely used options in cell biology are sometimes deemed inadequate. Below I present a list of technical issues in imaging strategies during the course of a systems biology project, from the initial design to data analysis and interpretation. The particular choices made for each stage can significantly affect whether the monitoring results represent physiological behaviors of the molecular network of interest.

### 2.1. Unnatural Activity of Fluorescent Fusion Proteins from the Transgene

All imaging studies rely on the assumption that the visualized protein is a reasonably accurate surrogate of the endogenous protein. For most live cell imaging approaches, the measured signal comes from fluorescent proteins, typically expressed from a transgene encoding the fluorescent protein. Even in the more tempered situation where the transgene is stably integrated in the genome, the expression pattern of the fluorescent protein varies depending on the copy number and the genomic contexts. Single cell cloning allows sampling and selection of cells with a most desirable expression pattern which recapitulates that of the endogenous gene. The following are some measures that help guide the design of the expression construct and subsequent selection of single cell clones. These precautions would avoid unintended re-wiring of the regulatory network and ensure visualization of the natural activity of the molecular system.

#### 2.1.1. Expression Level

**Figure 1:**
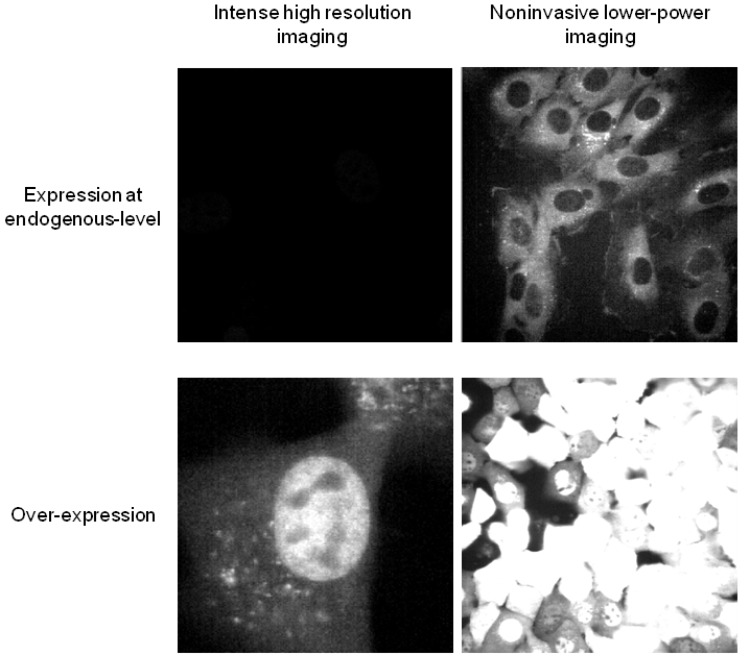
Lower expression requires a different set of imaging parameters. Upper panels show microscopy images of cells expressing GFP from an endogenous gene locus. Lower panels convey cells expressing a large amount of GFP under Tet-off self-amplification. Left panels are images obtained using an acquisition setting for high spatial resolution subcellular imaging, often used in cell biology. Right panel images were obtained using a setting with minimal illumination (lower power laser, shorter exposure, *etc*.) and maximal signal integration (zoom-out, thicker optical slice, *etc*.). All images were taken from Zeiss LSM 5 Live.

Tools for expressing DNA constructs have traditionally been geared toward high expression levels. In many applications, a protein is ectopically over-expressed or expressed at a sufficiently high level that permits high intensity signal from the fluorescent proteins (necessary for the optical resolution of many microscopy methods in cell biology; Figure 1). However, a goal in many systems biology studies is to express the fluorescent protein at levels comparable to the natural counterpart. Often the protein of interest in systems biology is part of a molecular network with nonlinear interactions and feedback loops. Increasing the level of one protein can lead to altered behaviors of the molecular system. Therefore, it would be inappropriate to use common promoters such as CMV that produce high protein levels. If the fluorescent protein is expressed in the presence of the endogenous gene, then snapshot measurements from single cells (e.g., immunofluorescence) or from populations (e.g., western blot) could be performed to compare the levels of the transgene and the endogenous gene [3,4]. If the transgene is expressed in cells lacking the expression of the endogenous gene (knock-out or knock-in cells), then the expression level can be compared to wild-type cells [5]. Such knock-out or knock-in systems eliminate the concern about interference or competition between unlabeled endogenous proteins and the fluorescently labeled molecules.

The requirement to keep the expression level of the fluorescent protein in the endogenous range must also be balanced with the competing need for visualizing the fluorescence and achieving a useful signal-to-noise ratio. For some proteins, it may be challenging to satisfy these two opposite criteria (Figure 1). The cellular stress induced by repeated image acquisition is another limiting factor in enhancing the image quality by adjusting the microscope setting (see Section 2.2, Section 2.3, Section 2.4, Section 2.5).

#### 2.1.2. Stimulus-Dependent Regulation of Expression

**Figure 2:**
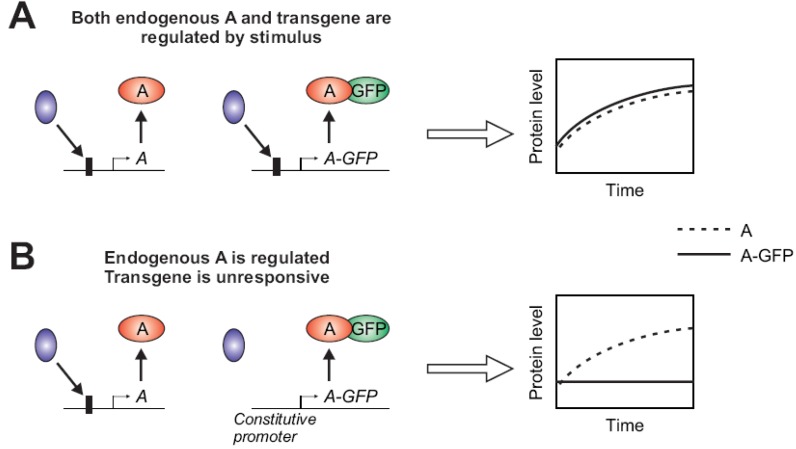
(**A**,**B**) The gene of interest “A” is transcriptionally activated by stimulus-induced binding of a transcriptional regulator (blue) at the promoter region (a box upstream of *A*). Subsequently, its protein level increases over time after stimulation. The expression of the GFP-fused version of A may not be properly regulated depending on the presence (**A**) or absence (**B**) of the specific binding sites in the promoter upstream. When the GFP-fusion does not retain a similar regulation of expression, the visualized temporal profile (solid curve) may deviate from the natural counterpart (dashed curve).

An often overlooked parameter when designing the construct for a fluorescent protein is the regulatory content of the transgene. Depending on the choice of promoters or other regulatory DNA elements (e.g., binding sites for specific transcription factors) included in the construct, the fluorescent protein may be either constitutively expressed (more common) or induced/repressed by stimuli. In systems biology studies of stimulus-responsive signaling dynamics, the protein of interest (to be fluorescently labeled) may be direct targets of relevant signaling. For example, the protein may be a component of a transcriptional feedback or feed-forward network. Since real-time dynamic activity can greatly depend on feedback loops, it is important to capture stimulus-dependent regulation of the expression. Hence, when the gene is known to be regulated by the stimuli under study, it is recommended that the sequence upstream of the fluorescent protein-coding DNA contain a promoter from the native locus including a minimal set of sites that confer appropriate responsiveness (Figure 2). However, it may not always be obvious whether the gene of interest is regulated or which regulatory sites are required, due to complexity of transcriptional regulation [6]. For these reasons, systems biology groups have increasingly chosen a natural promoter [3,7] over a constitutive promoter [8,9] to drive the expression of the fluorescent protein. Recently, BAC-based constructs have been preferred [10,11] with the expectation that they mimic the natural regulation of the endogenous gene more closely. New genome editing methods have also been developed, which permit more efficient generation of knock-in cell lines by insertion of a fluorescent transgene exactly at the endogenous locus [12,13].

#### 2.1.3. Fluorophore Interfering with Protein Function

The final product from the construct must behave as the (unlabeled) native protein in terms of subcellular localization, oligomerization, degradation, interactions with other biomolecules (DNA, RNA, protein, ATP, metabolites, *etc*.). Since the visualized molecule is often a fusion of the protein of interest and a fluorescent protein attached at the *N*- or the *C*-terminus, it may not only have a larger size but also have altered molecular function. Each important property needs to be confirmed by comparing to that of the endogenous protein. For example, certain fluorophores tend to form dimers or tetramers *in vivo*, inducing improper behaviors of the fusion protein. Using structural knowledge if available, the position of the fluorophore can be placed far from important functional domains in order to preserve protein-protein interaction or substrate recognition.

### 2.2. Optimal Spatial Resolution

In general, higher spatial resolution imaging requires more intense illumination and therefore delivers more photo-toxicity to cells. Especially for long duration imaging, it is essential to minimize cellular stress induced by repeated illumination. Another contributing factor to photo-toxicity is the choice of microscopy platforms, e.g., confocal versus wide-field imaging. Photo-damaged cells may escape notice in the absence of immediate morphological phenotypes such as altered cell motility, cessation of cell division, or apoptosis. To determine the maintenance of cell health over the entire course of imaging, the viability and behavior of imaged cells can be compared to those from cells that received less intense or no imaging. The need for little or no photo-damage must be counter-balanced with the ability to extract useful information from each image frame (Section 2.6). The setting for frame acquisition should have minimal spatial resolution and appropriate coverage (objective lens, pixel size, zoom, *etc*.) that still allows subsequent quantitative analysis (with a sufficient number of pixels per cell, for example). Such an optimal setting may be achieved far from what is conventionally used in routine cell biological applications, again requiring special attention (Figure 1).

### 2.3. Imaging Duration and Temporal Resolution

The length of the time course for live cell imaging is usually estimated initially from available experimental data generated by other methods. However, the necessary length that spans the entire course of response after stimulation, for example, sometimes turns out to be longer than anticipated. Such instances may arise when live imaging reveals a subpopulation of cells that have distinct late-stage activities. 

**Figure 3:**
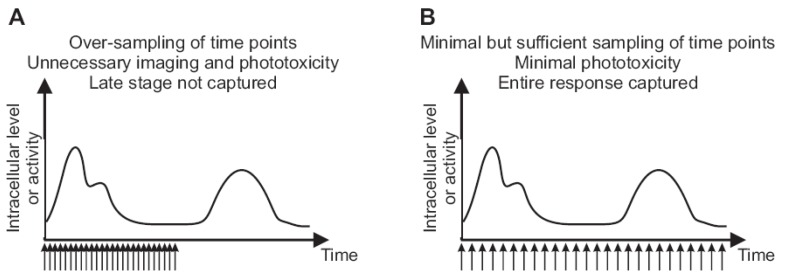
The frequency of imaging and duration of time lapse capture influence cell viability and coverage of relevant dynamic patterns. (**A**) Too frequent acquisition of time points for which the images are acquired without adding information about the dynamic pattern. Frequent illumination causes toxicity or cell stress that alters the physiological behavior of cells during monitoring. The duration of imaging is not long enough to capture late-stage activity. (**B**) The same number of time points as in (**A**) are distributed over a longer time course. The temporal resolution is still sufficient to capture fluctuations, while producing little or no photo-toxicity. The duration of monitoring is long enough to cover the complete response.

Determination of the imaging duration affects the frequency of time lapse imaging, *i.e.*, the interval between successive imaging. There are multiple factors to consider when deciding the time interval (Figure 3). First, the timescale of the biological process under investigation sets a minimum required sampling of time points for imaging. Many molecular networks that are examined in systems cell biology involve relatively slow (minutes to hours) processes like degradation, transcriptional activation and subsequent protein synthesis, and fluctuations of protein concentration in a subcellular compartment through transport mechanisms. Second, photo-toxicity from repeated imaging must be avoided in order to obtain physiological results. As discussed in Section 2.2, photo-damaged cells may not present obvious signs and may go undetected unless careful control experiments are performed. By varying the time interval between successive imaging, one can determine the maximum frequency of imaging that cells tolerate under the experimental conditions. If possible, another factor to consider is cell tracking during image analysis. Tracking the same cell over time lapse imaging is essential for live cell imaging and preferably carried out automatically using a matching algorithm (often by the nearest-centroid method). Such an automatic detection becomes more challenging for long time intervals between successive time frames, where a cell can appear to abruptly “jump”, crawl over another cell, disappear out of view, die, or divide.

### 2.4. Autofocus

A reliable autofocus mechanism is lacking in many “standard” cell biology microscopes, since in most applications the user focuses the objective lens at the sample and collects images on the spot with manual operations. However, many systems biology investigations require long-term image acquisition that can last hours or days (Section 2.3). It is not feasible to stand by the microscope over the entire time course and manually adjust focus whenever image sharpness is visually deemed poor. It is tempting to circumvent this problem simply by obtaining multiple z stacks with the expectation that at least one stack would capture the relevant cellular fluorescence signal. Such an attempt is equally frustrating to deal with because of the added photo-toxicity and complicated image stack selection and quantification. Stabilizing the stage temperature is helpful for reducing focus drift but does not eliminate focus fluctuations entirely. There are several different autofocus strategies and commercially available mechanisms. A free microscope control software package such as µManager [14] may be used to configure a custom autofocus solution on certain hardware. One can choose a mechanism that minimizes additional illumination and photo-damage to cells. 

### 2.5. Importance of Cell Culture Conditions during Setup and Image Acquisition

A unique requirement for live cell imaging is to maintain uncompromised incubation conditions for the cells during microscopy. There should be little or no unintended perturbation of cell microenvironment, from the moment of taking the dish or chamber of cells from the incubator until the end of imaging. To this end, it is preferable to minimize the physical effects (shifts in temperature, pH, mechanical impact and shaking, *etc*.) of moving the cells from the incubator to the microscope stage, by having the incubator close to the microscope room or by allowing some recovery/settling time after mounting the cell dish on the microscope before the start of imaging. For a prolonged duration of imaging, it is crucial to keep a stable culture condition with constant temperature (37 °C for most mammalian cells), CO2 level, and humidity, which usually requires a special inner chamber holding the cell dish and a larger temperature-stabilizing box surrounding the microscope stage. Other substitute devices (stage-mounted chamber unit without an enclosing box, objective heater, hot air blower, *etc*.) are generally inadequate for maintaining stable temperature due to a steep temperature gradient from cells to the microscope components in room temperature.

### 2.6. Quantitative Analysis to Extract Relevant Information from Imaging Data

Once a complete series of time lapse image frames is acquired, extraction of meaningful information from the data requires quantitative analysis algorithms specific to the investigation. For certain systems biology projects, widely available commercial or public software may not address the analysis needs. In case a custom analysis algorithm needs to be implemented, a computing environment such as MATLAB is a powerful tool that allows programming and quantification while providing a suite of built-in commands that handle basic image-related operations. There are also freely available software tools such as ImageJ [15], CellProfiler [16], and PhenoRipper [17] that allow users to perform many useful image analysis tasks. At a minimum, single cell analysis entails the segmentation of cell boundaries for each image frame acquired.

If the project calls for quantification of signal from a subcellular compartment (e.g., nucleus or plasma membrane), further segmentation is necessary to identify the compartment from each cell. If a compartment-specific label (whether genetically encoded or chemically introduced) is used and imaged, the analysis pipeline can be nearly automated with only occasional human interventions for spotting errors [18]. However, in live cell imaging, the use of a compartment-specific label is sometimes not feasible for practical reasons (reduction of an imaging channel that could otherwise be used for monitoring another protein, additional illumination and photo-toxicity, cell viability and/or the biological process affected by the dye, *etc*.). In the absence of an objective label, segmentation of the compartment relies on imaging data itself and becomes a much harder computational task, necessitating extensive, if not entirely, manual segmentation [5]. Therefore, this can be a major determinant of how labor-intensive the analysis procedure would be.

### 2.7. Sufficient Number of Single Cells for Statistically Significant Analysis Results

Single cell observations typically have cell-to-cell variability. Therefore, it is important to determine what outliers are and what are frequently observed behaviors or whether there are subpopulations of cells with distinct behaviors that have functional relevance [19]. Some studies have utilized microfluidic systems that enhance the throughput of live cell examination [20]. But certain microfluidic settings have limitations regarding one or more of the requirements discussed in the sections above. For example, the objective may be low resolution or the incubation may not be optimal in a particular setup [21,22].

### 2.8. Comparison with a Computational Model of the Molecular Network

If essential components of the molecular network and their interactions are known, a mathematical model can be constructed to represent the individual reactions. Computer simulations can be performed to generate useful hypotheses prior to live cell observations. The initial modeling effort may guide experimental design by suggesting non-intuitive behaviors to look for. The experimental observation may confirm such hypotheses or reveal different results which motivate subsequent model revisions. Such an iterative process of modeling and experimentation is a hallmark of systems biology investigations and accelerates knowledge discovery.

## 3. Conclusions

Successful applications of live cell imaging in systems cell biology projects are still technically demanding (Table 1), despite offering tremendous benefits and potential advancements [1,2,3,5,7,8,18,23,24,25,26]. This checklist was compiled from the lessons personally learned in recent years as well as from successes, pitfalls, and limitations observed from the literature. Particular configurations that optimize individual aspects may depend on the cell type under study. For example, a new cell line may have a different sensitivity to photo-toxicity, or the cells may have different size and shape characteristics that require altered spatial resolution. I hope that this checklist will nevertheless be a useful reference that dissects different parameters for consideration and that it will facilitate wider dissemination of quantitative live cell imaging approaches in the systems biology community.

**Table 1:** Necessary skills and resources required for quantitative live cell imaging.

Sections	Multidisciplinary expertise	Hardware	Software
2.1: Fluorescent protein behavior	Molecular biology, biochemistry	n.a.	n.a.
2.2–2.4: Microscopy	Cell biology, biophysics, optics	Microscope control system	Time series, stage control, autofocus
2.5: Cell physiology	Cell biology	Incubation control system	n.a.
2.6: Image analysis	Math/physics	High performance computer	Image segmentation, tracking
2.7: Population sampling	Statistics	High performance computer	Statistical analysis
2.8: Network modeling	Math/physics/engineering	High performance computer	Mathematical modeling
